# Author Correction: Giant room temperature anomalous Hall effect and tunable topology in a ferromagnetic topological semimetal Co_2_MnAl

**DOI:** 10.1038/s41467-020-18687-z

**Published:** 2020-09-16

**Authors:** Peigang Li, Jahyun Koo, Wei Ning, Jinguo Li, Leixin Miao, Lujin Min, Yanglin Zhu, Yu Wang, Nasim Alem, Chao-Xing Liu, Zhiqiang Mao, Binghai Yan

**Affiliations:** 1 Department of Physics and Engineering Physics, Tulane University, New Orleans, LA 70118 USA; 2 Department of Condensed Matter Physics, Weizmann Institute of Science, Rehovot, 7610001 Israel; 3 Department of Physics, Pennsylvania State University, University Park, State College, PA 16802 USA; 4 Superalloys Division, Institute of Metal Reseach, Chinese Academy of Sciences, 110016 Shenyang, China; 5 Department of Materials Science and Engineering, Pennsylvania State University, University Park, State College, PA 16802 USA

**Keywords:** Topological insulators, Topological insulators

Correction to: *Nature Communications* 10.1038/s41467-020-17174-9, published online 10 July 2020.

The original version of the Supplementary Information associated with this Article contained an error in Supplementary Figs. 5a and 6 in which the longitudinal resistivity data of the I//(001) sample was incorrectly given as the resistance data. In the correct version of Supplementary Figs. 5a and 6, the resistance data has been converted to resistivity by multiplying a geometric factor of 0.667. The HTML has been updated to include a corrected version of the Supplementary Information.

