# Supplementary material for: Giant room temperature anomalous Hall effect and tunable topology in a ferromagnetic topological semimetal Co2MnAl
Source: Nat Commun. 2020 Jul 10;11:3476. doi: 10.1038/s41467-020-17174-9 (PMC7351740; doi:10.1038/s41467-020-17174-9)
Supplement: Supplementary file 1 — Supplementary Information [file 41467_2020_17174_MOESM1_ESM.pdf]

Supplementary Information
Giant room temperature anomalous Hall effect and tunable topology in a ferromagnetic topological semimetal Co_2MnAl

Peigang Li,[1,*] Jahyun Koo,[2,*] Wei Ning,[3,†] Jinguo Li,[4] Leixin Miao,[5] Lujin Min,[3,5] Yanglin Zhu,[3,1] Yu Wang,[3,1] Nasim Alem,[5] Chao-Xing Liu,[3] Zhiqiang Mao,[3,1,‡] and Binghai Yan[2,§]

[1]*Department of Physics and Engineering Physics, Tulane University, New Orleans, LA 70118, USA*
[2]*Department of Condensed Matter Physics, Weizmann Institute of Science, Rehovot 7610001, Israel*
[3]*Department of Physics, Pennsylvania State University, University Park, Pennsylvania 16802, USA*
[4]*Superalloys Division, Institute of Metal Reseach, Chinese Academy of Sciences, Shenyang 110016, China*
[5]*Department of Materials Science and Engineering, Pennsylvania State University, University Park, Pennsylvania 16802, USA*

Supplementary Table I. **Summary of anomalous Hall conductivity (σ_{AHE}) and anomalous Hall angle (AHA) of currently studied magnetic materials.** Some AHA values are calculated by using the data from the references. Only the maximum values of AHA are adopted here. For Co_2MnGa and Co_2MnAl, the largest values at low temperature are also presented for comparison.

Materials	σ_{AHE}	AHA=Θ_{AH}	Temperature	Reference
	$\Omega^{-1}cm^{-1}$	(%)	K	
GdPtBi	110	16	10	Ref. 1
Fe_3Sn_2	1100	1.1	2	Ref. 2
MnSi	150	3.8	5	Ref. 3
Co_2FeAl	120	1.3	300	Ref. 4
Co_2FeSi	208	1.8	300	
$Co_3Sn_2S_2$	1130	19.8	120	Ref. 5
Mn_3Sn	100	3.2	100	Ref. 6
Fe_3GeTe_2	540	8.6	10	Ref. 7
$L1_0$-FePt film	1250	3.3	300	Ref. 8
Mn_5Ge_3	860	6.1	2	Ref. 9
MnGa film	288	5.7	300	Ref. 10
Fe (f)	1134	2.6	75	Ref. 11
CuZnCrSe	500	1.0	5	
$Fe_{0.28}TaS_2$	336	3.7	2	Ref. 12
$SrRuO_3$ film	200	1.0	5	Ref. 13
Mn_2Ru_xGa	220	7.7	300	Ref. 14
SmFe(f)	317	4.8	300	Ref. 15
Mn_3Ge	450	5.0	2	Ref. 16
TbCo film	800	3.2	300	Ref. 17
GaMnAs film	147	6.6	10	Ref. 18
Co_2MnGa	1100	12	300	Ref. 19
	2000	10	5	
**Co_2MnAl**	**1150~1277**	**15.3~21.2**	**300**	This work

* These two authors contributed equally
† wvn5038@psu.edu
‡ zim1@psu.edu
§ binghai.yan@weizmann.ac.il

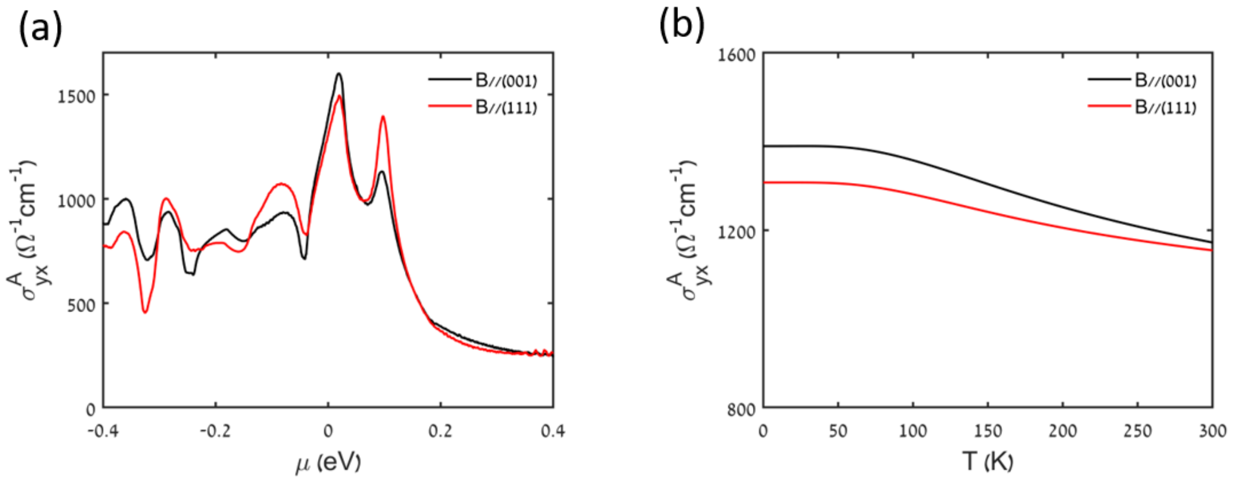

Supplementary Figure 1. **Compare the [001] and [111] directions.** Calculated anomalous Hall conductivity with respect to the chemical potential (a) and the temperature (b) for the magnetization along [001] (black) and [111] (red) directions. The $\sigma^A_{\rm yx}$ of [001] drops faster than that of [111], which is caused by the temperature-broadening in the Fermi-Dirac distribution.

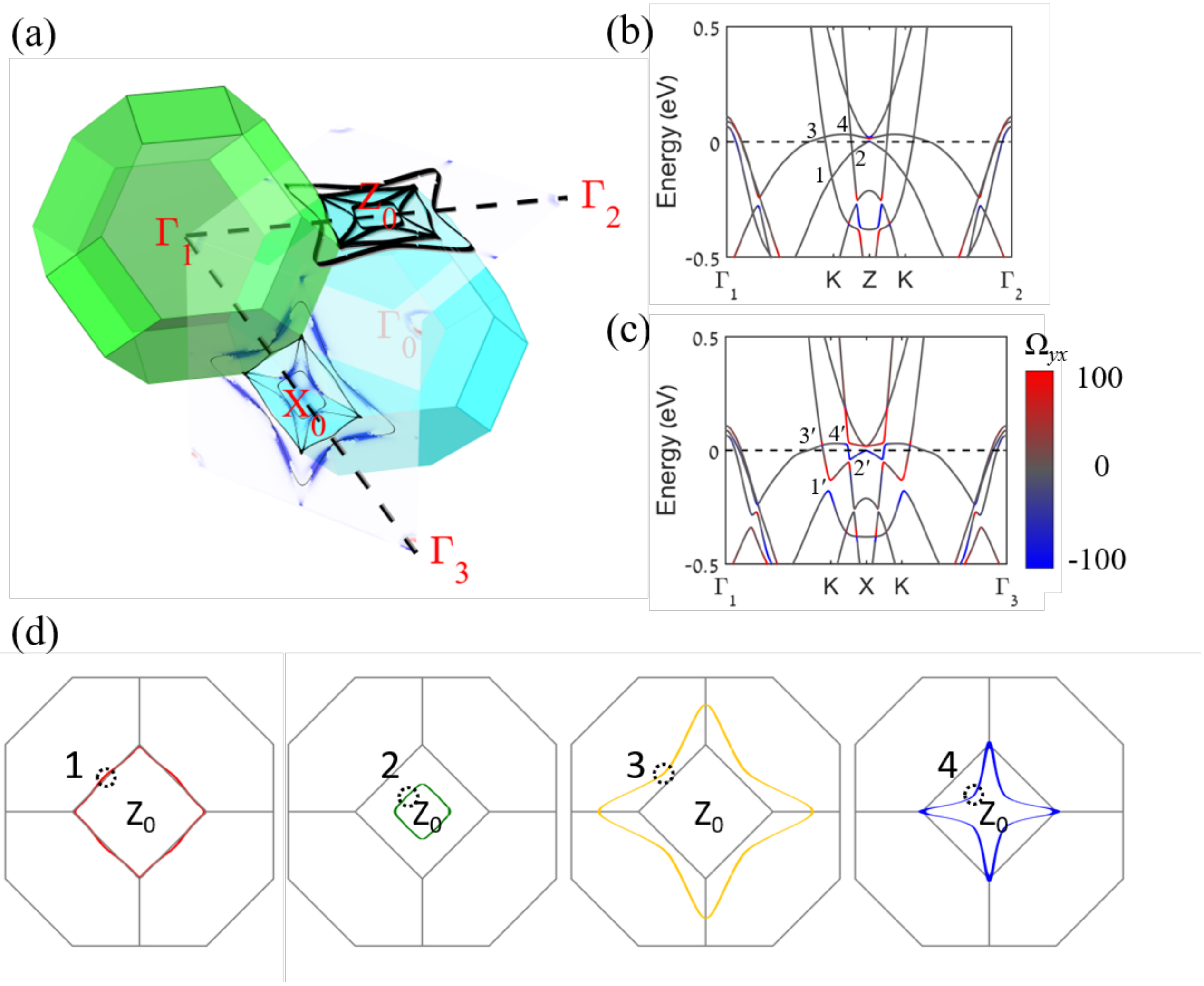

Supplementary Figure 2. **Band structure, Berry curvature and nodal rings.** (a) The Berry curvature at charge neutral point with gapped nodal rings in the X_0 plane and gappless nodal rings in the Z_0 plane. The magnetization axis is along the [001] direction. Green FCC Brillouin zone is the second Brillouin zone to the first one (blue). (b) Calculated band structure with Berry curvature from Γ_1 to Γ_2 as shown by the dashed line in (a). We label four crossing-points near the Fermi energy by #1-4. They are part of the nodal rings in the Z_0 plane and protected by the (001) mirror symmetry. These nodal rings does not generate the Berry curvature. (c) Calculated band structure with Berry curvature from Γ_1 to Γ_3 as shown by the dashed line in (a). All four cross-points (noted as # $1' - 4'$) are gapped and produce huge Berry curvature. (d) Each nodal ring in the Z_0 plane. Here we explain more detailed results of the [001] magnetization. The top two valence bands and bottom two conduction bands cross each other, resulting in four crossing points, as shown in (b). The equivalence of FCC Brillouin zone at the $\mathbf{k}_z = 0$ and $\mathbf{k}_z = \pi$ planes can be found in the (a). As the result of the magnetization, M_x or M_y mirror symmetry is broken and the nodal rings in the $\mathbf{k}_{x,y} = 0$ plane are gapped out and produce huge Berry curvature. Meanwhile, nodal rings in the $\mathbf{k}_z = 0$ plane are preserved by the M_z symmetry and generate zero Berry curvature. Nodal rings in different planes are inter-connected to each other. For example, in Fig. 3 in main text, nodal rings at the Z plane is locked to nodal rings in the X plane from another Brillouin zone. Since the Nodal lines has energy dispersion, we can find four peaks near Fermi energy relate with energy dispersion (Fig.3). Peak A is from gapped nodal ring #1, Peak B and C are from gapped nodal rings #2-3 and peak D from gapped nodal ring #4. The peaks arise when the Fermi energy shift cross the SOC gap of each gapped nodal rings. In addition, we also check spatial Berry curvature contribution in the first Brillouin zone. As expected, nothing can be found far from the nodal rings regions.

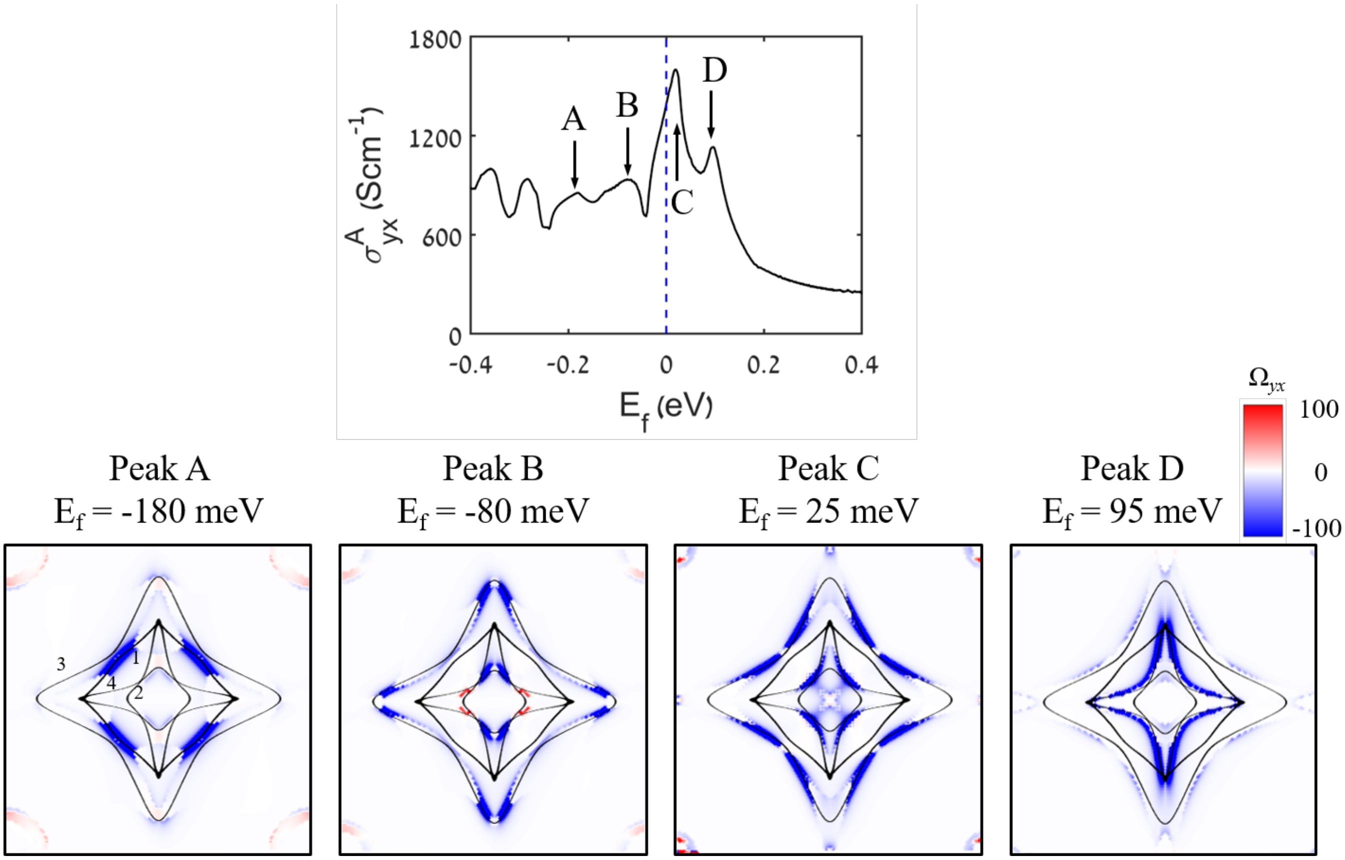

Supplementary Figure 3. **The anomalous Hall conductivity ($\sigma^A_{\mathbf{yx}}$) and nodal rings.** The calculated anomalous Hall conductivity with respect to the Fermi energy. The magnetization axis is along [001]. The charge neutral point is set to zero. Near the charge neutral point, there are four peaks of σ^A_{yx} noted as A,B,C and D. We show the gapped nodal rings in the $\mathbf{k}_\text{x} = 0$ plane (see Fig. 2 in main text) with the Berry curvature (Ω_{yx}) in the low panels. The σ^A_{yx} is mainly contributed by the gapped nodal rings. The peak C, which is the most relevant to the experiment, is induced mainly by the ring #3 and also slightly by the ring #2. Peak A is due to nodal ring #1, peak B due to #2 and slightly also #3 and peak D due to #4.

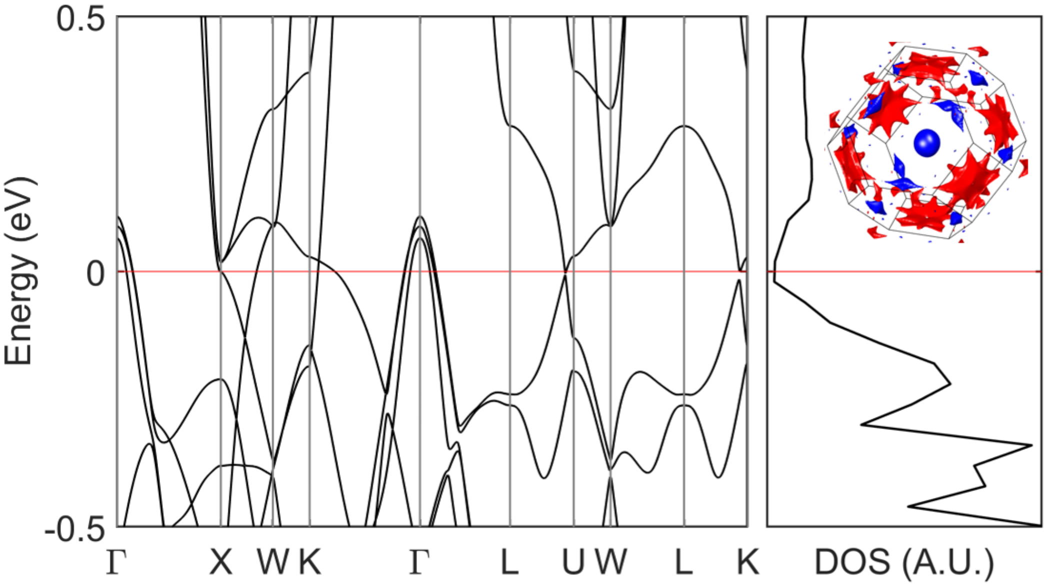

Supplementary Figure 4. **The calculated band structure of Co_2MnAl in the full Brillouin zone and the density of states (DOS).** The density of state near Fermi energy is large and exhibits an metallic feature. Inset in the density of state panel shows Fermi surfaces at charge neutral point in the FCC Brillouin zone.

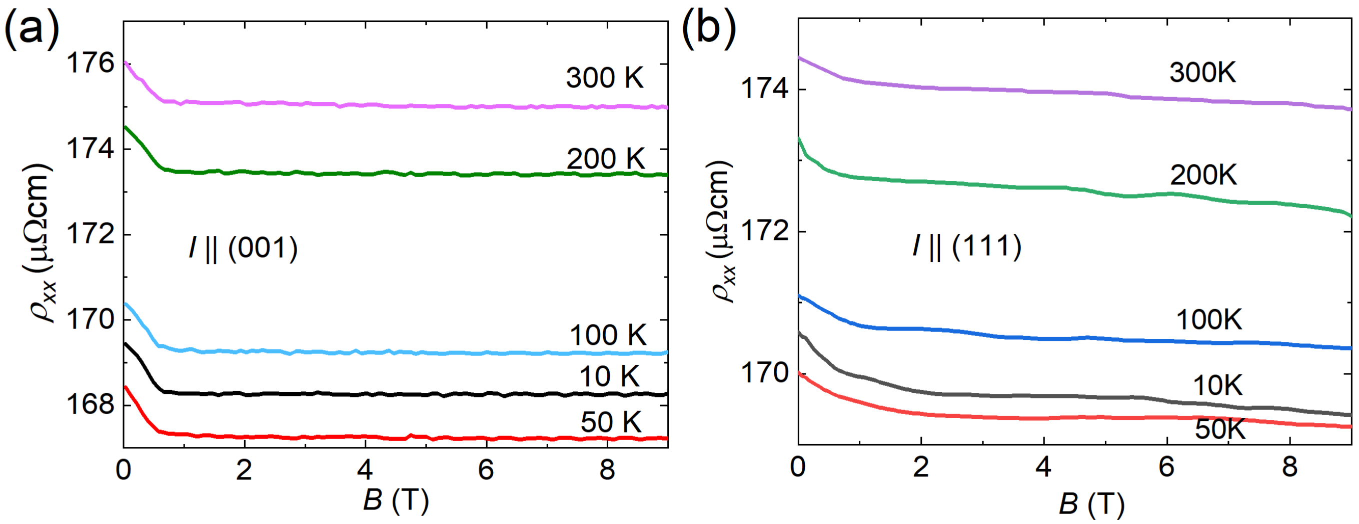

Supplementary Figure 5. **Magnetic field dependence of longitudinal resistivity ρ_{xx} for two samples with $I \parallel (001)$ and $I \parallel (111)$, respectively.**

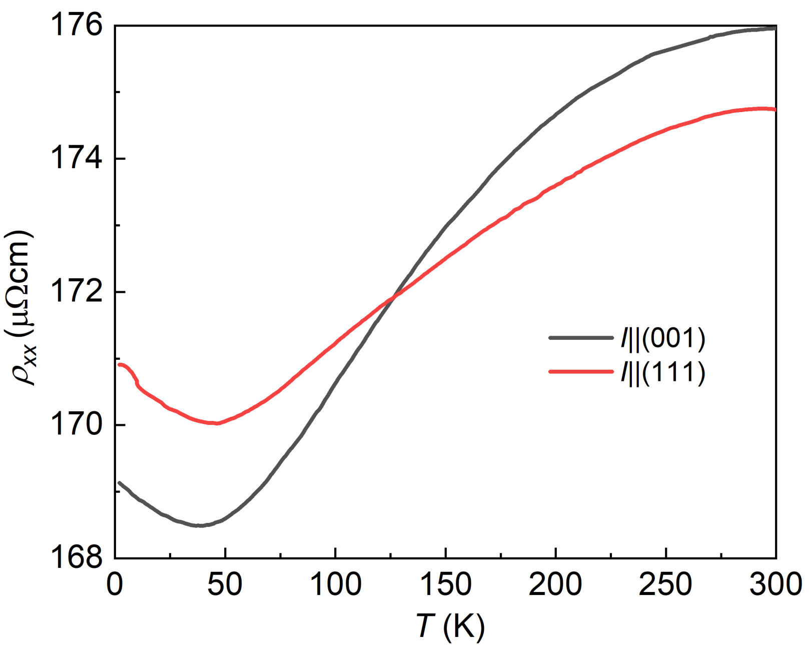

Supplementary Figure 6. **Temperature dependence of resistivity for two samples with $I \parallel (001)$ and $I \parallel (111)$.**

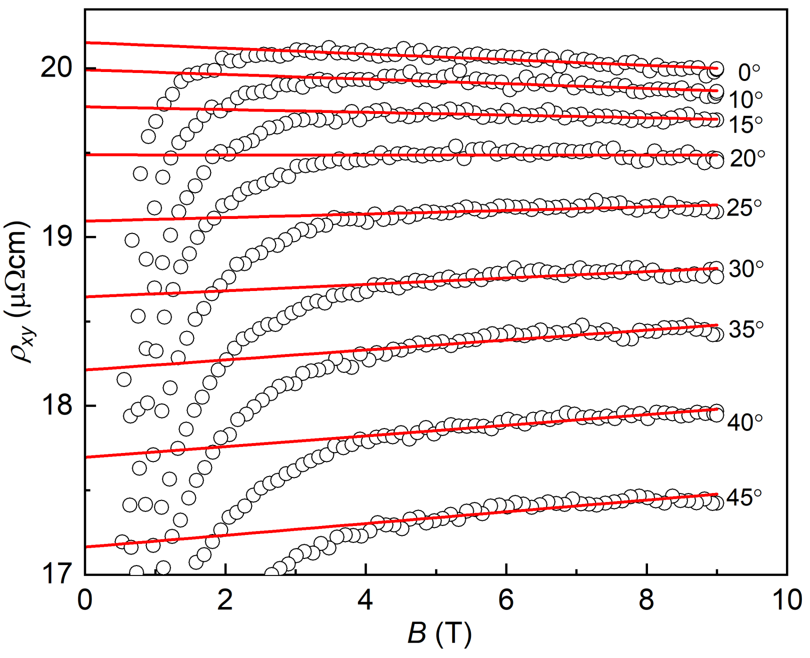

Supplementary Figure 7. **Magnetic field dependence of Hall resistivity ρ_{xy} under various orientation angles of magnetization (see the middle right inset in Fig. 2a) at 5K for the samples with $I \parallel (001)$**

I. SUPPLEMENTARY REFERENCES

1. Suzuki, T. et al. Large anomalous Hall effect in a half-Heusler antiferromagnet. Nat. Phys.12, 1119-1123 (2016).
2. Ye, L. et al. Massive Dirac fermions in a ferromagnetic kagome metal. Nature 555, 638 (2018).
3. Manyala, N. et al. Large anomalous Hall effect in a silicon-based magnetic semiconductor. Nat. Mater. 3, 255 (2004).
4. Imort, I. M. et al. Anomalous Hall effect in the Co-based Heusler compounds Co2FeSi and Co2FeAl. J. Appl. Phys. 111, 07D313 (2012).
5. Liu, E. et al. Giant anomalous Hall effect in a ferromagnetic kagome-lattice semimetal. Nat. Phys. 14, 1125–1131 (2018).
6. Nakatsuji, S. et al. Large anomalous Hall effect in a non-collinear antiferromagnet at room temperature. Nature 527, 212 (2015).
7. Kim, K. et al. Large anomalous Hall current induced by topological nodal lines in a ferromagnetic van der Waals semimetal. Nat. Mater. 17, 794-799 (2018).
8. Yu, J. et al. Magnetotransport and magnetic properties of molecular-beam epitaxy L10 FePt thin films. J. Appl. Phys. 87, 6854-6856 (2000).
9. Zeng, C. et al. Linear magnetization dependence of the intrinsic anomalous Hall effect. Phys. Rev. Lett. 96, 037204 (2006).
10. Wu, F. et al. Electrical transport properties of perpendicular magnetized Mn-Ga epitaxial films. Appl. Phys. Lett. 96, 042505 (2010).
11. Miyasato, T. et al. Crossover behavior of the anomalous Hall effect and anomalous Nernst effect in itinerant ferromagnets. Phys. Rev. Lett. 99, 086602 (2007).
12. Dijkstra, J. et al. Band-structure calculations of Fe1/3TaS2 and Mn1/3TaS2 , and transport and magnetic properties of Fe0.28TaS2. J. Phys.: Condens. Matter 1, 6363 (1989).
13. Fang, Z. et al. The anomalous Hall effect and magnetic monopoles in momentum space. Science 302, 92-95 (2003).
14. Thiyagarajah, N. et al. Giant spontaneous Hall effect in zero-moment Mn2RuxGa. Appl. Phys. Lett. 106, 122402 (2015).
15. Kim, T. et al. Spontaneous Hall effect in amorphous Tb–Fe and Sm–Fe thin films. J. Appl. Phys. 89, 7212-7214 (2001).
16. Nayak, A. K. et al. Large anomalous Hall effect driven by a nonvanishing Berry curvature in the noncolinear antiferromagnet Mn3Ge. Sci. Adv. 2, e1501870 (2016).
17. Kim, T. W. and Gambino, R. J. Composition dependence of the Hall effect in amorphous TbxCo1-x thin films. J. Appl. Phys. 87, 1869-1873 (2000).
18. Pu, Y. et al. Mott relation for anomalous Hall and Nernst effects in Ga1-xMnxAs ferromagnetic semiconductors. Phys. Rev. Lett. 101, 117208 (2008).
19. Sakai, A., et al. Giant anomalous Nernst effect and quantum-critical scaling in a ferromagnetic semimetal. Nat. Phys. 14, 1119-1124 (2018).
